# Sequence Type 4821 Clonal Complex Serogroup B *Neisseria meningitidis* in China, 1978–2013

**DOI:** 10.3201/eid2106.140687

**Published:** 2015-06

**Authors:** Bingqing Zhu, Zheng Xu, Pengcheng Du, Li Xu, Xiaofang Sun, Yuan Gao, Zhujun Shao

**Affiliations:** State Key Laboratory for Infectious Disease Prevention and Control, Beijing, China (B. Zhu, Z. Xu, P. Du, Z. Shao);; National Institute for Communicable Disease Control and Prevention, Chinese Center for Disease Control and Prevention, Beijing (B. Zhu, L. Xu, X. Sun, Y. Gao, Z. Shao);; Collaborative Innovation Center for Diagnosis and Treatment of Infectious Diseases, Hangzhou, China (Z. Shao)

**Keywords:** Neisseria meningitidis, serogroup B, serogroup C, sequence type 4821, ST-4821 clonal complex, clonal complex 4821, CC4821, capsule switching, China, epidemiology, bacteria, phylogenetic analysis, surveillance, recombination events, epidemic

## Abstract

Serogroup B *Neisseria meningitidis* strains belonging to sequence type 4821 clonal complex (CC4821), a hyperinvasive lineage first identified for serogroup C in 2003, have been increasingly isolated in China. We characterized the outer membrane protein genes of 48 serogroup B and 214 serogroup C strains belonging to CC4821 and analyzed the genomic sequences of 22 strains. Four serogroup B strains had porin A (i.e., PorA), PorB, and ferric enterobactin transport (i.e., FetA) genotypes identical to those for serogroup C. Phylogenetic analysis of the genomic sequences showed that the 22 CC4821 strains from patients and healthy carriers were unevenly clustered into 2 closely related groups; each group contained serogroup B and C strains. Serogroup B strains appeared variable at the capsule locus, and several recombination events had occurred at uncertain breakpoints. These findings suggest that CC4821 serogroup C *N. meningitidis* is the probable origin of highly pathogenic CC4821 serogroup B strains.

## Abstract 2

These highly invasive strains probably originated from CC4821 serogroup C strains.

*Neisseria meningitidis* bacteria are a leading cause of bacterial meningitis and other serious invasive bacterial infections. Among the 12 identified serogroups, A, B, C, Y, W, and X are responsible for most invasive meningococcal diseases. The geographic distribution and epidemic capabilities of *N. meningitidis* differ according to serogroup (*1*). On the basis of the epidemiology of *N. meningitidis*, many countries have included different formulations of the meningococcal vaccine in their routine immunization programs (*2*–*4*). These vaccines have significantly reduced the incidence of meningococcal diseases (*5*,*6*). However, in several countries, the introduction of vaccines targeting specific serogroups may have led to the replacement of vaccine serogroups by other, nonvaccine, serogroups (*7*–*11*). Serogroup replacement can occur as a result of capsule switching (*3*,*8*,*12*–*19*) or as a result of importation of a serogroup meningococcus from other regions (*20*).

In the past century in China, most meningococcal epidemics were caused by strains of *N. meningitidis* that belonged to sequence type 1 (ST-1) and ST-5 clonal complexes (CC1 and CC5, respectively) (*21*). Therefore, beginning in the early 1980s, a polysaccharide vaccine against serogroup A was incorporated into the routine immunization program. Use of this vaccine led to a significant decrease in the incidence of meningococcal diseases (*22*). However, in 2003, a serogroup C outbreak caused by a CC4821 strain was reported in China; this clonal lineage had not been detected in other countries (*23*). CC4821 corresponding to serogroup C has subsequently become one of the dominant lineages in China (*21*). To combat this serogroup replacement, several vaccines were developed against serogroup C or serogroups A and C. During 2005–2010, subsequent to the time when serogroup C and A *N. meningitidis* infections had been prevalent, cases caused by serogroup W strains belonging to CC11 began increasing in China (*24*,*25*). In addition to these 3 serogroups, serogroup B strains have been isolated from patients and healthy carriers. Serogroup B strains showed high genetic diversity and were usually associated with sporadic infections (*21*). Some of the prevalent clonal lineages that are common in many countries (e.g., CC32 and CC41/44) (*26*) are rarely isolated in China (*21*). However, CC4821 became a dominant lineage among serogroup B strains since they were first identified in 2005. When we retrospectively studied the strains in our collection, CC4821 strains were isolated as early as in 1978, and the lineage included serogroup B and C strains (*27*). Nevertheless, few CC4821 strains were isolated during 1970–1980, and no CC4821-related outbreaks were identified during that time (Z. Shao, unpub. data).

Capsular switching between *N. meningitidis* serogroups B and C is frequently observed (*3*,*7*,*8*,*12*,*16*–*19*); therefore, serogroups B and C most likely have similar DNA sequences in the capsule locus, leading to increased horizontal DNA transfer between these serogroups (*16*). We propose that capsular switching occurred between the CC4821 serogroup B and C *N. meningitidis* strains. To elucidate the relationship between them, we investigated the epidemiology of CC4821 serogroup B strains, characterized the outer membrane protein (OMP) genes of these strains, and analyzed the genome sequences and capsule locus sequences of specific strains.

## Materials and Methods

### Meningococcal Meningitis Surveillance in China

A population-based surveillance system for meningococcal meningitis exists throughout China. Provincial Center for Disease Control and Prevention (CDC) staff routinely collect strains suspected to be *N. meningitidis* on the basis of morphologic and biochemical characteristics, and they periodically conduct surveys of *N. meningitidis* carriers for outbreak investigation, surveillance, and research purposes. If no strain is isolated, clinical specimens are collected by the provincial CDC. The strains and specimens are sent to the China CDC national reference laboratory for identification, or they are tested at the provincial CDC, and results are sent to China CDC. Our laboratory identifies strains and performs multilocus sequence typing (MLST) on confirmed *N. meningitidis* strains.

### Meningococcal Strains and DNA Preparation

Forty-eight serogroup B and 214 serogroup C *N. meningitidis* strains previously assigned to CC4821 were included in this study. These strains were collected from 20 provinces in China during 1978–2013. Among the 48 serogroup B strains, 9 were from patients and 39 were from asymptomatic carriers. Among the 214 serogroup C strains, 91 were from patients and 123 were from asymptomatic carriers. One strain was identified as serogroup B by PCR; serogroups for the other strains were determined by slide agglutination with specific rabbit antisera (Remel Europe Ltd, Kent, UK) (Technical Appendix Table 1).

The selected strains were propagated on single plates containing Columbia agar in a 5% CO_2_ atmosphere at 37°C for 18 h. Genomic DNA was extracted by using the Wizard Genomic DNA Purification Kit (Promega, Madison, WI, USA) according to the manufacturer’s instructions.

### Sequencing of OMP Genes

The porin A (*porA*), *porB*, and ferric enterobactin transport (*fetA*) genes were amplified from freshly prepared DNA. The PCR and sequencing were performed as previously described (*28*–*30*).

### Genome Sequences of Meningococcal Strains

Eight serogroup B and 14 serogroup C *N. meningitidis* CC4821 strains were sequenced by constructing 2 paired-end libraries with average insert lengths of 500 bp. The sequences were generated by using an Illumina HiSeq 2000 sequencing platform (Illumina, San Diego, CA, USA) and assembled into contigs and scaffolds by using SOAP*denovo*, release 1.04 (http://soap.genomics.org.cn/soapdenovo.html). Genes were predicted by using Glimmer (*31*) with default parameters and then annotated by sequence comparisons with nucleotide and nonredundant protein sequence databases and the SwissProt (http://web.expasy.org/docs/swiss-prot_guideline.html) database by using BLAST (http://blast.ncbi.nlm.nih.gov) with an e-value of 1e−5. The genome sequence data obtained in this study were submitted to GenBank under the accession numbers JMBH00000000, JMCN00000000–JMCZ00000000, JMDA00000000–JMDH00000000. The complete reference genome sequences of multiple *N. meningitidis* strains and 1 *N. lactamica* strain were downloaded from the Completed Genomic Sequence section of the publicly available Entrez Genome database (http://www.ncbi.nlm.nih.gov/genomes/static/EG_T.html).

### Phylogenetic Analysis of *N. meningitidis* Genome Sequences

All 22 CC4821 genomes and 14 reference *N. meningitidis* genomes were used to construct a phylogenetic tree, and the genome of *N. lactamica* strain 020-06 was used as the outgroup. We identified the core genes in these 37 genomes in 2 steps. First, we used OrthoMCL (http://orthomcl.org/orthomcl/) to cluster all genes into orthologous groups and then selected the groups that were shared by all 37 genomes. Second, we removed the orthologous groups associated with known mobile genetic elements, such as genomic islands, phages, and transposons. The remaining orthologous groups were considered to be core genes. For all 37 genomes, the amino acid sequences of the core genes were concatenated, and multiple sequence alignments were performed by using MUSCLE (http://www.ebi.ac.uk/Tools/msa/muscle/). We then constructed a phylogenetic tree using the neighbor-joining method with MEGA4 (http://www.megasoftware.net/).

### Identification and Analysis of Capsule Locus

The contigs containing capsule locus sequences were compared with the *N*. *meningitidis* complete reference genome sequences by using blastn (http://blast.ncbi.nlm.nih.gov) to identify orthologous sequences and determine their levels of similarity. For contigs with a gap within the UDP-glucose 4-epimerase (*galE*) gene, PCR and Sanger sequencing were performed to close the gap. The capsule locus between genes transcriptional accessory protein (*tex*) and *galE* of CC4821 serogroup B strains were then aligned with the corresponding sequences from all serogroup B complete genomes by using MUSCLE version 3.6. On the basis of the alignment results, we used the genome with the highest level of similarity with CC4821 serogroup B strains as the reference sequence in subsequent analyses. To reveal the relationship between CC4821 serogroups B and C, we aligned the capsule locus genes from all study strains with those of serogroup C isolate 053442 (ST-4821) and the selected serogroup B reference strain.

### Identification of Recombination Breakpoints

We compared the capsule locus between genes *tex* and *galE* from study and reference strains by using MUSCLE. We analyzed recombination events within the capsule locus sequences by using different methods in RDP (Recombination Detection Program) version 4 beta 27 (http://web.cbio.uct.ac.za/~darren/rdp.html) with default parameters.

## Results

### Epidemiology of CC4821 Serogroup B Strains

**Figure 1:**
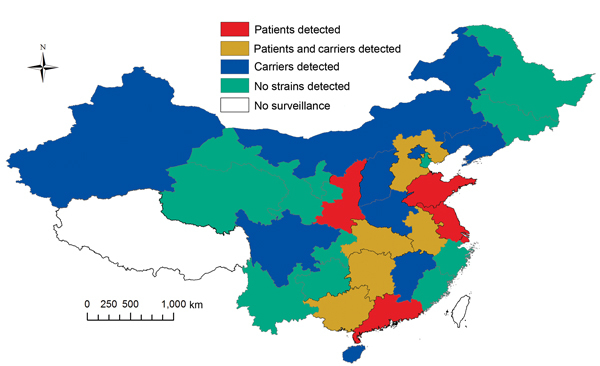
Distribution of *Neisseria meningitidis* sequence type 4821 clonal complex (CC4821) serogroup B strains in China, 1978–2013. Invasive strains were detected in 5 provinces (red), carriage strains were detected in 9 provinces (blue), and invasive and carriage strains were detected in 5 provinces (gold). Regions where CC4821 strains were not found or where surveillance is not conducted are also shown.

During the 1970s and 1980s, a total of 12 CC4821 serogroup B and C strains (6 from each serogroup) were collected from 6 provinces in China: Hebei, Henan, Jiangxi, Liaoning, Shanxi, and Shanghai. During March 2005–March 2013, *N. meningitidis* strains belonging to CC4821 serogroup B were isolated from the cerebrospinal fluid or blood samples of meningococcal patients in 10 provinces and from pharyngeal swab specimens from healthy carriers in 9 other provinces in China. These 19 provinces represent diverse geographic and climate conditions, and the cases were not related. (Figure 1)

### OMP Genotype Profiles

The *porA* gene was sequenced for all studied strains. The genotype profiles revealed a high degree of diversity even among strains with identical STs. Serogroup B *N. meningitidis* strains had 26 PorA genotypes, and serogroup C strains had 16. Among these, 8 genotypes were detected among both serogroup B and C strains, representing 35.4% of serogroup B and 77.6% of serogroup C strains. P1.7-2, 14 was the predominant genotype in serogroups C (55.6%) and B (12.5%). Two combinations of ST and PorA genotype were observed in serogroup C and B strains: ST-4821: P1.7-2, 14 and ST-4821: P1.20, 23-1. In total, 4 serogroup B strains had combination genotypes that were the same as those for serogroup C strains (Technical Appendix Table 2). To elucidate the relationship between the serogroup B and C strains, we sequenced the *porB* and *fetA* genes for strains with the combination genotype ST-4821: P1.7-2, 14 or ST-4821: P1.20, 23-1. The sequencing analysis showed that all 4 serogroup B strains had identical PorB and FetA genotypes (3-48 and F3-3, respectively), which were also the main PorB and FetA genotypes in the serogroup C strains.

### Phylogenetic Analysis of Genome Sequences

**Figure 2:**
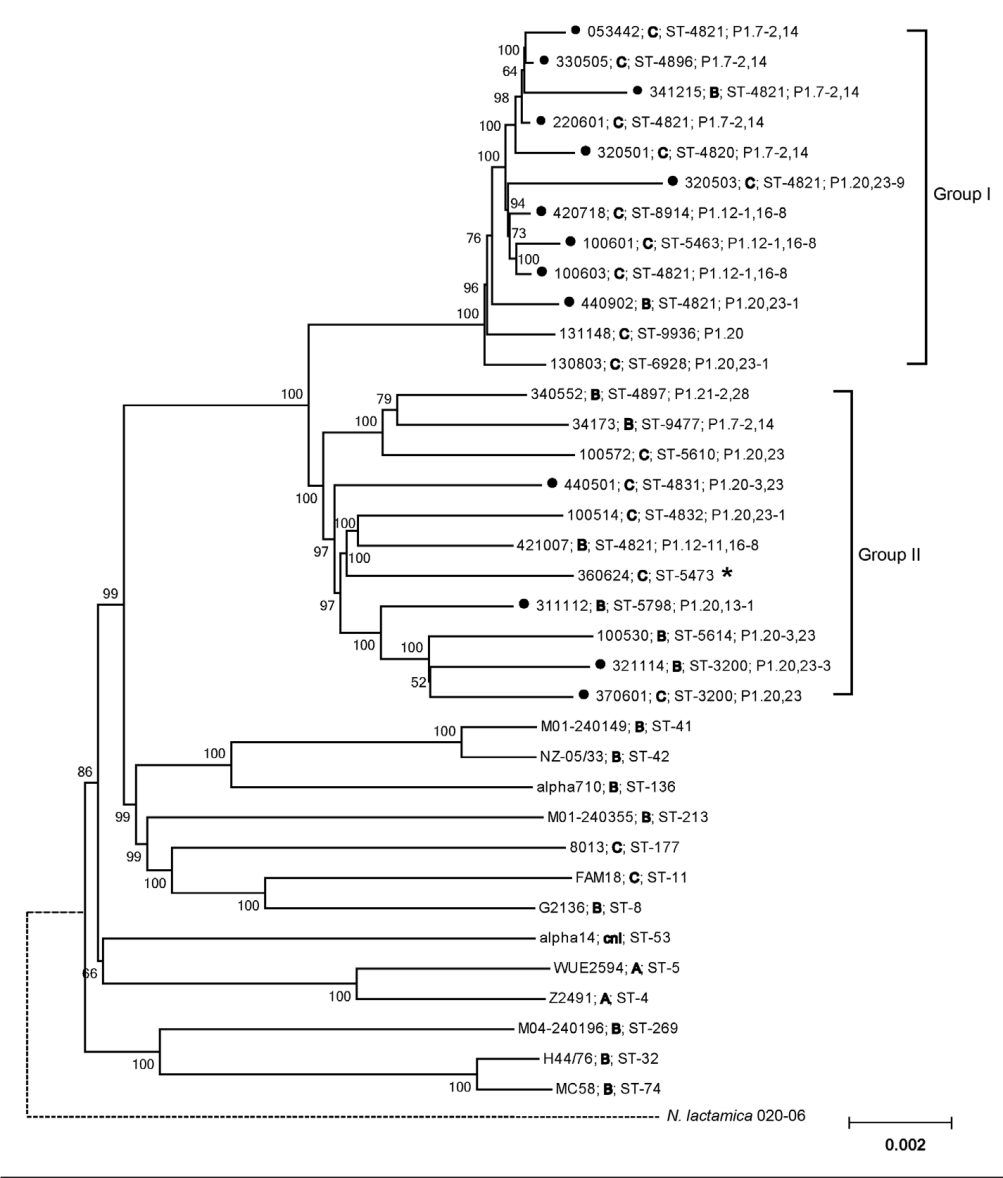
Phylogenetic analysis of genome sequences for *Neisseria meningitidis* strains. With the exception of reference strain 053442 (serogroup C, sequence type 4821), all strains in groups I and II were sequenced in this study. The strain identification number, serogroup (in boldface), sequence type, and porin A type are shown for each sequence. Bootstrap values are listed at nodes. Black dots preceding identification numbers indicate strains isolated from patients. The dotted line between *N. lactamica* 020-06 and *N. meningitidis* represents a distance not to scale. The star indicates that the porin A gene was not detected by PCR or genome sequencing. Scale bar indicates amino acid substitutions per site.

A total of 1,200 core genes (385,358 aa, corresponding to 50.9% of the genome of serogroup B *N. meningitidis* strain MC58) were selected for the phylogenetic analysis as described in Materials and Methods. The neighbor-joining phylogenetic tree reconstructed from the concatenated amino acid sequences of these genes showed that the 22 CC4821 strains were clustered into 2 closely related groups (groups I and II), which were distantly related to the *N. meningitidis* reference strains belonging to other CCs (Figure 2). Both groups contained serogroup B and serogroup C strains, although there were more serogroup C strains in group I and more serogroup B strains in group II. Each group consisted of 11 strains, but there were more invasive strains in group I (n = 9) than in group II (n = 4). The reference strain 053442, which was isolated from a patient and belonged to CC4821, was clustered with group I. Considering the genetic distances between the strains, the strains in group II were less closely related to each other than those in group I.

### Analysis of Capsular Locus

The capsule locus sequences between genes *tex* and *galE* were retained from 20 sequenced CC4821 genomes (12 serogroup C and 8 serogroup B). For 2 strains, there were gaps within *galE* or *ctrA* gene.

**Figure 3:**
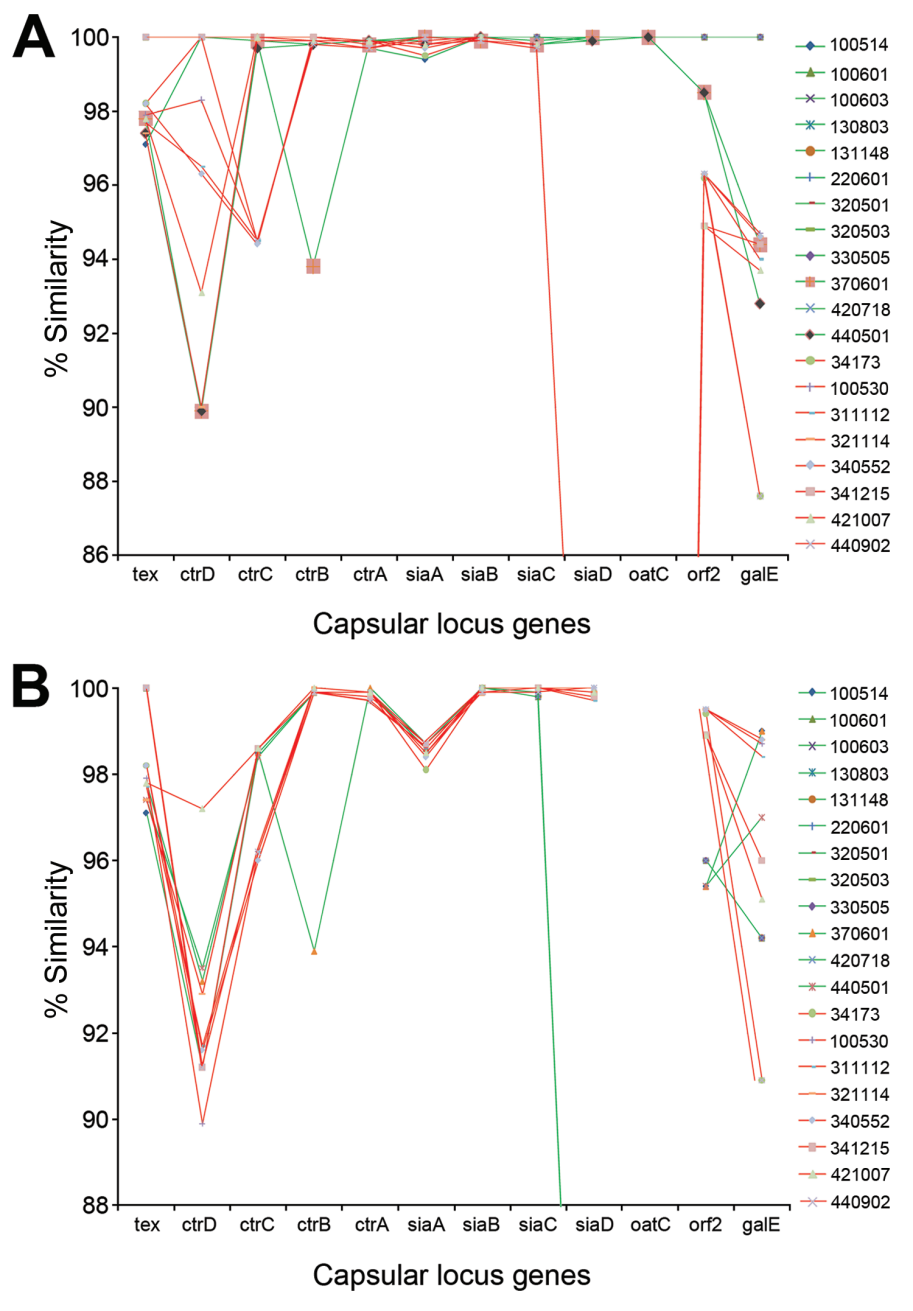
Analysis of capsular locus sequences from *Neisseria meningitidis* strains belonging to the sequence type 4821 clonal complex (indicated on right). A) Similarity between the capsular locus genes of study strains and those from reference strain 053442 (serogroup C). B) Similarity between the capsular locus genes of study strains and those from reference strain H44-76 (serogroup B). Green lines indicate serogroup C strains; red lines indicate serogroup B strains.

The DNA sequences of the capsular locus were compared with the homologous gene clusters of the reference strains (053442, serogroup C, ST-4821; H44-76, serogroup B, ST-32) (Figure 3). All capsular locus genes of 9 serogroup C strains (group I) were 99.9%–100% identical to that for reference strain 053442. The other 3 serogroup C strains (group II) were less similar to 053442 at the genes upstream of polysialic acid capsule export outer-membrane lipoprotein (*ctrA*) and downstream of sialic acid synthase (*siaC*). All serogroup B strains had polysialyltransferase (*siaD*) genes that were distinct from that for reference strain 053442 and did not contain the O-acetyltransferase (*oatC*) gene between genes *siaD* and open-reading frame 2. With the exception of 1 serogroup B strain (341215, in group I), which shared high similarity (99.8%–100%) with 053442 at the genes upstream of *siaD*, the serogroup B strains had low similarity at *ctrC* and preceding genes. Serogroup B and C strains shared <99% similarity with strain H44-76 at genes *tex*, *ctrD*, *ctrC*, *siaA*, *open-reading frame 2*, and *galE*. However, *siaD* genes of the serogroup B strains and H44-76 shared >99.7% similarity.

**Figure 4:**
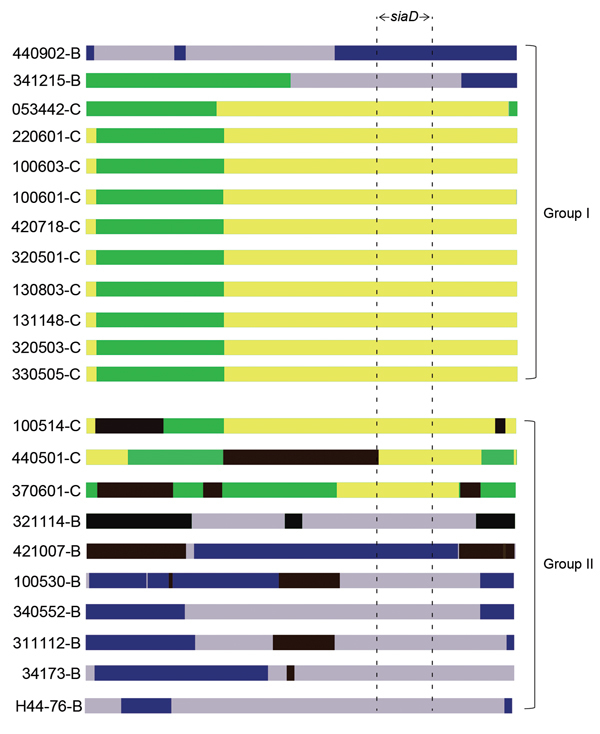
Analysis of the recombination events *Neisseria meningitidis* strains belonging to the sequence type 4821 clonal complex (strain numbers and serogroup are shown on the left). The result was from analysis using the 3seq (*33*) method in RDP (http://web.cbio.uct.ac.za/~darren/rdp.html). Group I and group II refer to the groups in Figure 2. Green regions represent serogroup C–specific sequences; yellow regions represent the recombination within serogroup C; gray regions represent serogroup B–specific sequences; blue regions represent the recombination within serogroup B; black regions represent the recombination between serogroup B and C. Location of the polysialyltransferase gene (*siaD*) is shown.

We used RDP, GENECONV (http://www.math.wustl.edu/~sawyer/geneconv/), BootScan (*32*), and the 3seq method (*33*) to analyze the recombination events within the capsule locus, but failed to obtain consistent results about the breakpoint. Nevertheless, all the results indicated that different and multiple events had occurred at the capsule locus within and among strains (Figure 4). Compared with strains belonging to group II, those belonging to group I (genome-based phylogenetic tree) had fewer recombination events, and most of the events were within the same serogroup.

## Discussion

Phylogenetic analysis of the *N. meningitidis* core genome amino acid sequences showed that all 22 sequenced CC4821 strains were closely related, irrespective of serogroup, ST, and PorA type, which indicated capsular switching between serogroups C and B. However, serogroups B and C were detected in both phylogenetic groups, so the direction of capsule switching remains to be determined (*34*). The multiple recombination events may have occurred within the capsule locus, which explains why we could not define the recombination breakpoints at the capsule locus. The present evidence is not sufficient to confirm whether capsular switching occurred from serogroup C to B or vice versa. Further study is required to examine how the capsular switching occurred.

In previous studies of *N. meningitidis* capsule switching, MLST and OMP genotyping were used to characterize the relationship between the new variants and the candidate parental organisms (*3*,*7*,*8*,*16*,*18*,*19*). If the molecular profiles (e.g., ST, PorA, and FetA) are identical or highly similar between 2 different serogroups, capsule switching is inferred. In our study, only 4 ST-4821 serogroup B *N. meningitidis* strains were observed to share the same STs and OMP gene types (PorA, PorB, and FetA) with serogroup C *N. meningitidis*. However, analysis of the core genome sequencing results showed that all serogroup B strains, even those with specific profiles, are closely related to serogroup C strains. Horizontal DNA transfer occurs frequently between *N. meningitidis* strains. Recombination events involving the MLST locus and the *porA*, *porB*, and *fetA* genes would impede the analysis of capsule switching and might lead to false conclusions (*15*). An analysis based on genomic sequence can reveal the relationships among strains more accurately, so additional genome analysis is required to resolve the observed discrepancies.

CC4821 serogroup C *N. meningitidis* had been the dominant lineage in China for a decade (2003–2014), although the incidence of invasive disease remained at a moderate level (<0.1 case/100,000 population; Z. Shao, unpub. data) because of mass vaccination. The emergence and circulation of CC4821 serogroup B *N. meningitidis* might increase the incidence of invasive meningococcal diseases and even cause epidemics and outbreaks in China. This potential risk can be attributed to the CC4821 lineage itself and to the particularity of vaccine against serogroup B *N. meningitidis*. Since the first outbreak occurred in 2003, the CC4821 serogroup C *N. meningitidis* epidemic has rapidly involved most provinces of China. This epidemic indicates that CC4821 *N. meningitidis* had substantial ability to spread extensively and cause invasive disease. The core genome–based phylogenetic analysis showed that CC4821 strains from patients and healthy carriers were unevenly clustered into 2 groups, suggesting a difference in pathogenicity between these 2 groups of strains. Furthermore, group I, which possessed more patient-derived strains, was the most recent clade on the tree, and the strains in this group were more closely related to each other than those in group II, indicating that group I was a highly invasive sublineage of CC4821. Because of the emergence of serogroup B strains belonging to this highly invasive *N. meningitidis* CC4821 sublineage, we must remain alert for a potential epidemic. An effective protective vaccine specifically against the serogroup B capsule polysaccharide does not exist (*35*). Although several vaccines consisting of specific proteins have been licensed for use against serogroup B infection (*36*), their effectiveness varies by clonal lineage, and their effectiveness has not been studied in China. This critical public health concern highlights the need for further epidemiologic surveillance to monitor changes in the incidence of meningococcal disease caused by *N. meningitidis* CC4821 serogroup B and for improved public health disease control strategies in the future.

Circulation of the CC4821 clonal lineage has not been observed in other regions, even though it is hyperendemic in China. The reason for this limited distribution is not readily apparent, which highlights the need for continued surveillance. The virulence and pathogenic mechanisms of this newly identified hyperinvasive lineage are not well understood (*23*). Comparative genome analysis within CC4821 strains and those from other CCs may help to identify potential additional virulence factors of *N. meningitidis*. In China, mass vaccination against meningococcal disease targets only *N. meningitidis* serogroups A and C. Thus, monitoring the appearance and spread of CC4821 in other serogroups is important because capsule switching among *N. meningitidis* serogroups C, B, W, and Y has been observed in several countries (*14*,*16*).

**Technical Appendix.** Isolation year and location (province) and PorA genotypic profiles of *Neisseria meningitidis* strains belonging to the sequence type 4821 clonal complex, China.
