# Supplementary material for: Sequence Type 4821 Clonal Complex Serogroup B Neisseria meningitidis in China, 1978–2013
Source: Emerg Infect Dis. 2015 Jun;21(6):925–32. doi: 10.3201/eid2106.140687 (PMC4451889; doi:10.3201/eid2106.140687)
Supplement: Supplementary file 1 — Technical Appendix. Isolation year and location (province) and PorA genotypic profiles of Neisseria meningitidis strains belonging to the sequence type 4821 clonal complex, China. [file 14-0687-Techapp-s1.pdf]

Article DOI: http://dx.doi.org/10.3201/eid2106.140687

Sequence Type 4821 Clonal Complex Serogroup B *Neisseria meningitidis* in China, 1978–2013

Technical Appendix

Technical Appendix Table 1. Isolation year and location (province) of *Neisseria meningitidis* strains belonging to serogroup B sequence type 4821 clonal complex, China

	No. isolates													
Province	1978–1980	2000	2002	2003	2004	2005	2006	2007	2008	2009	2010	2011	2012	2013
Anhui						1							1	
Beijing						2								
Guangdong		1							1					
Guangxi						1						16		
Hebei													1	
Hubei											2			1
Jiangsu												1		
Jiangxi						1								
Shandong						2								1
Shanxi										11				
Shanghai								1				1		
Sichuan						1	2							

Technical Appendix Table2. The PorA genotypic profile of 262 *Neisseria meningitidis* strains belonging to clonal complex 4821, China, 2000–2013

Sequence type	PorA type profile	Serogroup, n
ST-4821	P1.7–2, 14*	B, 3; C, 113
	P1.20, 23–1*	B, 1; C, 7
	P1.20, 23–7*	C, 18
	P1.12–1, 16–8	C, 20
	P1.20, 9	C, 19
	P1.20, 23–6*	C, 6
	P1.20, 23–9	C, 2
	P1.18, 25–11	C, 1
	P1.20, 23*	C, 1
	P1.20–2, 23–9	C, 1

Sequence type	PorA type profile	Serogroup, n
	P1.12–11, 16–8	B, 2
	P1.20, 2	B, 1
	P1.20, 23–18*	B, 1
	P1.20, 23–3	B, 1
ST-3200	P1.20, 23*	C, 1
	P1.20, 23–3	B, 1
	P1.20, 14	B, 4
	P1.20–5, 14	B, 1
ST-5610	P1.20, 23*	C, 3
ST-3436	P1.12–12, 23–4	B, 2
	P1.20–3, 23–14	B, 1
ST-4897	P1.21–2, 28	B, 1
ST-5474	P1.18, 25*	B, 1
ST-5584	P1.20, 23–16	B, 1
ST-5614	P1.20–3, 23*	B, 2
ST-5618	P1.20–4, 23–2	B, 1
ST-5664	P1.20, 23–3	B, 1
	P1.18, 25*	B, 1
	P1.20, 23–1*	B, 1
	P1.20, 23–6*	B, 1
	P1.20, 23–7*	B, 1
ST-5798	P1.22, 23–9	B, 1
	P1.22–28, 14–22	B, 1
	P1.19, 13–13	B, 1
	P1.21–10, 16	B, 1
	P1.7–2, 14*	B, 1
	P1.20, 13–43	B, 1
	P1.20, 13–1	B, 1
ST-7298	P1.7–2, 14*	B, 1
ST-8687	P1.21–10, 16	B, 1
ST-8688	P1.21–10, 16	B, 1
ST-8690	P1.20, 23–3	B, 1
ST-8691	P1.20, 23–3	B, 1
ST-8697	P1.5–1, 23	B, 1
ST-9477	P1.7–2, 14*	B, 1
	P1.20, 23–18*	B, 2
ST-9478	P1.20–4, 23–6	B, 1
ST-9480	P1.22–28, 14–22	B, 1
ST-10164	P1.20, 23*	B, 1

Sequence type	PorA type profile	Serogroup, n
ST-4820	P1.7–2, 14*	C, 4
ST-4831	P1.20–3, 23*	C, 2
ST-4832	P1.20, 23–1*	C, 2
ST-4833	P1.18, 25*	C, 1
ST-4837	P1.18, 34	C, 1
ST-4896	P1.7–2, 14*	C, 1
ST-4980	P1.20, 23–7*	C, 1
ST-5081	P1.20, 23–2	C, 1
ST-5463	P1.12–1, 16–8*	C, 2
ST-5473	P1.22, 23	C, 1
	Other†	C, 1
ST-6928	P1.20, 23–1*	C, 2
ST-8914	P1.12–1, 16–8	C, 1
ST-9936	P1.20	C, 1
ST-9942	P1.7–2, 14*	C, 1

*PorA types shared by serogroup B and C strains.

†The *porA* gene was not detected by PCR and genome sequencing.
